# Global burden of type 2 diabetes attributable to secondhand smoke: a comprehensive analysis from the GBD 2021 study

**DOI:** 10.3389/fendo.2025.1506749

**Published:** 2025-04-29

**Authors:** Dongke Guo, Yanna Yu, Zhongxin Zhu

**Affiliations:** ^1^ Department of Science and Education, The First People’s Hospital of Xiaoshan District, Xiaoshan Affiliated Hospital of Wenzhou Medical University, Hangzhou, China; ^2^ Department of Public Health, The First People’s Hospital of Xiaoshan District, Xiaoshan Affiliated Hospital of Wenzhou Medical University, Hangzhou, China; ^3^ Department of Clinical Research, The First People’s Hospital of Xiaoshan District, Xiaoshan Affiliated Hospital of Wenzhou Medical University, Hangzhou, China

**Keywords:** global burden of disease, secondhand smoke, type 2 diabetes mellitus, socio-demographic index, disability-adjusted life years, estimated annual percentage changes

## Abstract

**Introduction:**

Secondhand smoke (SHS) exposure represents an underappreciated global health risk for type 2 diabetes mellitus (T2DM), with complex epidemiological implications.

**Methods:**

Leveraging the comprehensive Global Burden of Disease (GBD) 2021 dataset, we systematically evaluated the worldwide burden of type 2 diabetes mellitus attributable to secondhand smoke (T2DM-SHS) across 204 countries. The analysis encompassed both death and disability-adjusted life years (DALYs) across various genders, age groups, and 204 nations over the period from 1990 to 2021. We examined trends and socioeconomic impacts by analyzing age-standardized DALYs rates and estimated annual percentage changes, stratified by socio-demographic Index (SDI) quintiles.

**Results:**

The following changes occurred between 1990 and 2021: while age-standardized mortality rates decreased by 8.903% (95% UI: -16.824% to -1.399%), DALYs increased by 17.049% (95% UI: 9.065% to 25.557%). Age-stratified analysis revealed peak death in the 70–74 years group, with females experiencing highest DALYs in the 75–79 years group and males in the 90–94 years group. An inverted U-shaped relationship between SDI and disease burden emerged, with peak rates at moderate SDI levels.

**Discussion:**

Despite lowest burdens in high-income countries, disease dynamics were most complex in middle-range SDI countries, indicating that economic development does not linearly correlate with health outcomes. This comprehensive analysis unveils the multifaceted global landscape of T2DM-SHS, exposing critical disparities across gender, age, and socioeconomic contexts. The findings urgently call for targeted, context-specific public health interventions, particularly in low- and middle-income countries, to mitigate the escalating T2DM-SHS burden.

## 1 Introduction

Type 2 diabetes mellitus (T2DM) has been increasingly recognized by the World Health Organization (WHO) as a critical global health threat, characterized by chronic hyperglycemia resulting from progressive insulin resistance and pancreatic β-cell dysfunction (1, 2). Emerging evidence has highlighted secondhand smoke (SHS) exposure as a significant yet underappreciated risk factor for T2DM pathogenesis. Comprising a complex mixture of over 7,000 toxic compounds, SHS induces multifaceted pathophysiological alterations through intricate molecular mechanisms, including oxidative stress, chronic inflammation, endothelial dysfunction, and epigenetic modifications (3–6). Recent meta-analyses have demonstrated that SHS exposure increases the risk of developing T2DM by 22%, underscoring its substantial global health impact (3, 7, 8). The magnitude of this health burden is starkly illustrated by the 1.29 million deaths and 34.9 million disability-adjusted life years (DALYs) attributed to SHS exposure globally in 2021 (4).

Despite the established causal relationship between SHS exposure and T2DM, critical knowledge gaps remain in elucidating the nuanced interactions between demographic characteristics, socioeconomic indices, and type 2 diabetes mellitus attributable to secondhand smoke (T2DM-SHS) burden. Existing studies have limitations, particularly in isolating the independent effects of SHS on T2DM from other risk factors, as well as in conducting age- and gender-stratified analyses of disease burden over time across different socio-demographic Index (SDI) regions. The heterogeneity of these interactions presents multifaceted research challenges that warrant systematic investigation. Specifically, three key dimensions underscore the importance of this research (1): SHS exposure patterns exhibit significant variability across populations, with socioeconomic contexts potentially modulating both exposure intensity and metabolic vulnerability; (2) intervention effectiveness demonstrates marked demographic-specific variations, necessitating precision-targeted public health strategies; and (3) evidence-based resource allocation requires comprehensive risk stratification of populations and regions most susceptible to T2DM-SHS interactions.

The complex interplay between these factors and their influence on T2DM-SHS burden remains poorly characterized, particularly in vulnerable populations and resource-limited settings. This knowledge gap significantly hampers the development of targeted interventions and effective policy measures. To address these critical research needs, we conducted a comprehensive analysis using the GBD 2021 dataset. Our study aims to: (1) quantify the global burden of T2DM-SHS from 1990 to 2021; (2) identify high-risk populations and regions that would benefit most from targeted interventions; and (3) provide evidence-based recommendations for developing future public health strategies and policies.

## 2 Methods

### 2.1 Data acquisition and sources

This study uses data from the GBD 2021 dataset, which is a comprehensive database that records the incidence, prevalence, and death rates of 371 diseases and injuries across 204 countries and regions. We focused on the burden of T2DM-SHS. Estimates were generated by sex and age (from birth to 95+ years, with 5-year intervals and a separate group for those over 95) and stratified by SDI, a composite measure of socioeconomic development.

The burden estimation process closely followed GBD 2019 methods, with updates detailed in the appendix. We analyzed death and DALYs associated with T2DM-SHS. Data were extracted using the Institute for Health Metrics and Evaluation (IHME) results tool (http://ghdx.healthdata.org/gbd-results-tool). The comparative risk assessment methodology for SHS followed previously published GBD protocols.

### 2.2 SDI analysis

The correlation between the SDI and the burden of T2DM-SHS was investigated by computing disease rates specific to each SDI stratum. The SDI categories, ranging from low to high, were employed to juxtapose the disease burden across various socioeconomic development levels. For the manipulation and visualization of data, the Dplyr and ggplot2 packages in R were utilized, providing a robust framework for analyzing and graphically representing the relationship between SDI and T2DM-SHS burden.

### 2.3 Geographic regions

The GBD 2021 study encompassed estimates for 204 countries and territories, which were aggregated into 21 regions and further categorized into seven super-regions. These GBD regions and super-regions are composed of geographically proximate countries and territories that exhibit epidemiological similarities and share comparable patterns in the distribution of causes of death. GBD 2021 study encompassed estimates for 204 countries and territories, which were aggregated into 21 regions and further categorized into seven super-regions. These GBD regions and super-regions are composed of geographically proximate countries and territories that exhibit epidemiological similarities and share comparable patterns in the distribution of causes of death.

### 2.4 Exposure to SHS case definition

SHS exposure was comprehensively defined as involuntary inhalation of environmental tobacco smoke from direct proximity to active smokers or in smoke-contaminated environments. In alignment with the GBD 2019 standardized methodology, exposure was quantified through a multi-dimensional assessment incorporating: (1) self-reported exposure duration and frequency, (2) biochemical markers of tobacco smoke metabolites (cotinine levels), and (3) environmental tobacco smoke concentration measurements. Participants were categorized into exposure levels based on validated thresholds: non-exposed (no reported or measured smoke contact), low-exposure (intermittent or occasional environmental tobacco smoke contact), and high-exposure (consistent and prolonged smoke environment interaction). The operational definition considered both domestic and occupational smoke exposure, accounting for variations in smoke density, duration, and proximity to active smokers. Exposure assessment utilized standardized questionnaires validated across diverse demographic and geographical contexts, ensuring robust and comparable measurement across different population subgroups (9).

### 2.5 SHS-attributable burden

Estimates of attributable burden for T2DM-SHS were established by multiplying the relevant cause measure by the PAF. All estimates for risk groupings or all risk factors combined are generated via an aggregation process that accounts for the fact that the effect of one risk factor might be partly or completely mediated through the effect of another. This mediation analysis is informed by individual-level data from prospective cohort studies on the joint effects of combinations of risk factors.

Age and gender specific PAF for SHS was calculated using the following standard formula:


$$\text{PA}{\text{F}}_{\text{SHS}}=\;{\text{P}}_{\text{SHS}}\;(\text{R}{\text{R}}_{\text{SHS}}-1)/[{\text{P}}_{\text{SHS}}\;(\text{R}{\text{R}}_{\text{SHS}}-1)]\;+\;1$$

GBD analytical method for estimating PAF has six analytical steps. (1) We included 19 risk–outcome pairs that met criteria for convincing or probable evidence on the basis of research studies. (2) Relative risks were estimated as a function of exposure based on published systematic reviews, GBD review and meta-regression. (3) Levels of exposure in each age-sex-location-year included in the study were estimated based on all available data sources using spatiotemporal Gaussian process regression, DisMod-MR 2.1, a Bayesian meta-regression method, or alternative methods. (4) We determined, from published trials or cohort studies, the level of exposure associated with minimum risk, called the theoretical minimum risk exposure level. (5) Attributable DALYs were computed by multiplying PAFs by the relevant outcome quantity for each age sex-location-year. (6) PAFs and attributable burden for combinations of risk factors were estimated taking into account mediation of different risk factors through other risk factors (9).

### 2.6 Statistical analysis

To quantify the burden of T2DM-SHS, we utilized age-standardized DALYs as a comprehensive measure of overall health loss. DALYs account for both YLDs and YLLs, with each DALYs representing the loss of one full year of healthy life. A linear regression model to analyze trends in age-standardized DALYs from 1990 to 2021 at national, regional, and global levels. The model used the natural logarithm of age-standardized rates (ASRs), following the equation:


ln(ASR) =α+βX +ϵ

where ‘X’ represents the calendar year. The Estimated Annual Percentage Change (EAPC) was used to quantify the average annual change in age-standardized rates over the study period. This method ensures that each observation contributes to the calculation of EAPCs providing estimates of long-term trends in disease burden indicators.

The EAPC and its corresponding 95% confidence interval (CI) using the formula:


EAPC = 100×(exp(β) − 1).

The trend was considered stable if the 95% CI of the EAPC included 0 (*P* ≥.05). An increasing trend was identified when both the EAPC and its 95% CI were >0, while a decreasing trend was noted when these values were <0. To explore the association between the SDI and the age-standardized DALYs due to T2DM-SHS, we employed a LOESS regression analysis.

## 3 Results

### 3.1 Global level burden and trends of T2DM-SHS

Our analysis revealed significant changes in the burden of T2DM-SHS exposure between 1990 and 2021. Globally, the age-standardized mortality rates (ASMR) per 100,000 population decreased from 0.956 (95% UI: 0.353-1.554) in 1990 to 0.870 (95% UI: 0.318-1.439) in 2021 (**Table 1**), representing a reduction of 8.903% (95% UI: -16.824% to -1.399%). This decline was accompanied by a negative EAPC of -0.487 (95% CI: -0.573 to -0.400), indicating a consistent downward trend in death over the study period.

**Table 1:** Death and DALYs of T2DM-SHS in 1990 and 2021 for both sexes and all regions.

	Death						DALYs					
	Number of cases, 1990	Age-standardised rate per 100 000 populaiong, 1990	Number of cases, 2021	Age-standardised rate per 100 000 populaiong, 2021	Percentage change 1990-2021 (%)	Age-standardised rate EAPCs	count, 1990	Age-standardised rate per 100 000 populaiong, 1990	Count, 2021	Age-standardised rate per 100 000 populaiong, 2021	Percentage change 1990-2021 (%)	Age-standardised rate EAPCs
Global	35471.020(13073.540-57576.039)	0.956(0.353-1.554)	73902.304(26909.119-122094.487)	0.870(0.318-1.439)	-8.903(-16.824--1.399)	-0.487(-0.573--0.400)	1489404.908(534931.753-2502172.670)	36.329(13.080-61.041)	3687910.196(1331071.650-6309283.637)	42.523(15.353-72.769)	17.049(9.065-25.557)	0.292(0.217-0.368)
SDI												
High	5232.532(1861.005-8778.941)	0.476(0.169-0.798)	5657.294(2007.785-9555.103)	0.264(0.093-0.441)	-44.596(-47.869--41.383)	-2.357(-2.578--2.135)	231306.828(80933.776-397810.258)	21.811(7.644-37.524)	746666.199(265993.277-1287452.656)	23.948(8.360-42.552)	9.797(-1.670-20.041)	-0.011(-0.140-0.118)
High-middle	7158.736(2639.875-11617.716)	0.785(0.288-1.280)	12913.561(4724.991-21620.810)	0.656(0.240-1.100)	-16.501(-24.875--8.014)	-0.688(-0.793--0.583)	326634.127(118117.479-555866.606)	32.379(11.660-55.216)	916789.495(333195.938-1571249.666)	39.185(13.911-67.640)	21.020(10.627-30.549)	0.442(0.345-0.538)
Middle	12467.297(4706.311-20051.170)	1.407(0.532-2.270)	28521.313(10504.117-47645.003)	1.129(0.418-1.886)	-19.771(-28.236--11.458)	-0.938(-1.028--0.847)	532755.147(192413.751-889001.025)	49.349(17.912-82.209)	1377250.925(495742.485-2341468.571)	49.910(18.018-84.670)	1.135(-6.450-8.879)	-0.240(-0.329--0.152)
Low-middle	8083.645(2947.864-13205.473)	1.534(0.558-2.504)	21633.759(7796.669-36404.175)	1.700(0.612-2.851)	10.842(-2.202-24.411)	0.278(0.220-0.336)	305941.959(112041.436-505774.317)	48.241(17.743-79.615)	226997.902(78073.205-388439.038)	61.369(22.403-104.560)	27.213(15.679-38.277)	0.684(0.646-0.721)
Low	2474.564(873.379-4172.811)	1.255(0.450-2.094)	5089.175(1760.357-8568.564)	1.166(0.401-1.977)	-7.150(-18.285-4.749)	-0.391(-0.485--0.297)	90641.717(32094.146-152704.886)	37.994(13.455-63.997)	416194.861(145795.140-736610.449)	40.909(14.124-69.828)	7.673(-2.764-18.412)	0.031(-0.051-0.113)
Regions												
East Asia	6060.760(2323.451-9865.630)	0.810(0.312-1.313)	13536.592(4895.437-22430.886)	0.655(0.238-1.084)	-19.173(-36.044-2.613)	-0.873(-1.104--0.642)	338313.651(126424.830-579779.823)	36.611(13.709-62.957)	889017.941(320148.328-1544586.439)	42.025(15.094-72.481)	14.787(0.021-29.201)	0.191(0.080-0.302)
Southeast Asia	4698.854(1669.882-7600.440)	2.051(0.737-3.313)	11789.586(4226.835-19559.497)	1.973(0.702-3.268)	-3.765(-17.925-11.472)	-0.282(-0.362--0.202)	168123.837(59737.701-274051.432)	63.347(22.512-102.621)	479429.074(175160.450-811219.864)	71.110(25.996-120.108)	12.255(-0.919-25.090)	0.148(0.071-0.224)
Oceania	169.266(57.680-286.272)	6.632(2.345-11.096)	484.907(165.287-824.772)	7.307(2.504-12.419)	10.185(-13.975-39.627)	0.210(0.143-0.276)	5789.257(1969.027-9942.384)	182.697(62.423-310.581)	19506.214(6735.500-32989.216)	233.426(81.072-396.470)	27.767(1.637-59.601)	0.697(0.636-0.757)
Central Asia	265.595(94.522-446.982)	0.570(0.203-0.958)	768.617(273.638-1307.856)	0.953(0.341-1.607)	67.054(44.395-90.792)	1.450(0.977-1.925)	13113.509(4617.468-22325.511)	26.544(9.342-45.178)	44864.428(16268.248-76850.605)	50.046(18.191-86.025)	88.538(71.844-105.071)	1.928(1.611-2.247)
Central Europe	1097.254(394.423-1821.886)	0.748(0.269-1.239)	1472.120(532.034-2501.018)	0.635(0.230-1.081)	-15.031(-22.050--6.238)	-0.349(-0.488--0.209)	52358.270(19294.922-89706.754)	34.872(12.889-59.738)	73426.082(26039.947-127879.323)	35.857(12.738-62.172)	2.824(-4.695-9.862)	0.199(0.129-0.269)
Eastern Europe	642.210(237.093-1055.268)	0.226(0.083-0.372)	1645.769(572.080-2734.390)	0.455(0.159-0.755)	101.256(74.812-128.059)	1.013(-0.121-2.159)	43767.767(15587.125-76172.868)	15.622(5.568-27.277)	82382.116(29163.130-144434.517)	24.574(8.723-43.252)	57.303(43.866-72.066)	0.932(0.673-1.192)
High-income Asia Pacific	1006.628(362.679-1650.531)	0.520(0.186-0.856)	712.926(250.311-1237.330)	0.139(0.050-0.239)	-73.236(-76.790--69.749)	-4.623(-4.818--4.427)	57836.590(20630.839-100695.262)	28.253(10.077-49.180)	86839.574(30175.797-158390.666)	25.696(8.910-46.832)	-9.051(-22.178-2.698)	-0.689(-0.852--0.525)
Australasia	86.459(30.800-144.509)	0.378(0.135-0.632)	98.166(33.551-167.467)	0.178(0.061-0.302)	-52.849(-59.456--46.280)	-2.949(-3.234--2.664)	3535.766(1241.560-6220.760)	15.561(5.458-27.508)	5816.138(2028.636-10339.997)	12.506(4.364-22.051)	-19.630(-31.389--8.280)	-0.988(-1.102--0.873)
Western Europe	2973.733(1066.391-4995.459)	0.507(0.182-0.851)	2077.059(721.010-3582.307)	0.196(0.068-0.333)	-61.452(-64.130--58.987)	-3.095(-3.260--2.930)	103817.151(37140.820-175919.597)	19.297(6.893-32.681)	123209.536(43218.594-218725.759)	16.839(5.815-29.939)	-12.738(-24.233--2.638)	-0.533(-0.627--0.440)
Southern Latin America	547.857(196.206-903.768)	1.226(0.438-2.022)	584.973(203.106-993.429)	0.659(0.230-1.119)	-46.197(-50.623--41.437)	-2.213(-2.480--1.945)	18497.103(6769.655-31102.333)	39.831(14.554-66.977)	31646.084(11302.083-55362.534)	37.493(13.369-65.588)	-5.869(-17.412-5.206)	-0.433(-0.601--0.265)
High-income North America	1510.955(529.431-2557.922)	0.437(0.153-0.739)	1553.317(541.687-2622.993)	0.245(0.085-0.411)	-43.953(-48.812--39.522)	-2.663(-3.104--2.219)	64768.624(21802.182-111524.760)	19.947(6.733-34.354)	127315.611(43388.686-227305.000)	22.320(7.656-39.643)	11.897(-0.840-24.434)	-0.049(-0.237-0.140)
Caribbean	432.429(152.466-716.441)	1.751(0.616-2.903)	532.980(180.144-920.343)	0.980(0.331-1.690)	-44.031(-51.586--35.965)	-2.241(-2.408--2.075)	15232.162(5507.133-25516.677)	58.007(20.991-97.107)	25754.857(8897.064-45320.982)	48.009(16.573-84.432)	-17.236(-26.309--7.641)	-0.974(-1.108--0.840)
Andean Latin America	125.410(42.201-211.487)	0.638(0.215-1.072)	262.464(85.140-470.833)	0.451(0.146-0.809)	-29.295(-42.876--14.430)	-1.592(-1.792--1.393)	4793.470(1573.045-8313.577)	22.216(7.307-38.610)	11878.214(3839.214-20601.241)	19.558(6.327-33.942)	-11.966(-25.514-1.404)	-0.883(-1.047--0.719)
Central Latin America	1998.065(736.364-3278.907)	2.571(0.948-4.223)	3172.609(1071.787-5227.511)	1.286(0.436-2.114)	-49.979(-56.118--43.171)	-2.640(-2.981--2.298)	76952.618(27899.497-128716.605)	86.900(31.599-145.595)	134527.501(46703.360-228884.205)	52.061(17.979-88.598)	-40.091(-46.401--33.646)	-2.082(-2.361--1.802)
Tropical Latin America	1791.644(665.419-2942.704)	2.158(0.795-3.558)	2430.550(870.717-4186.099)	0.963(0.345-1.654)	-55.387(-59.741--51.167)	-2.828(-2.986--2.670)	70163.010(25266.224-118350.447)	73.266(26.437-123.750)	106163.254(37431.043-184979.070)	40.629(14.322-70.813)	-44.546(-50.401--39.145)	-2.172(-2.290--2.053)
North Africa and Middle East	2382.278(858.011-3889.929)	1.702(0.614-2.793)	6930.191(2509.966-11708.149)	1.758(0.631-2.967)	3.316(-12.027-14.776)	0.422(0.259-0.586)	91454.032(32815.007-153554.153)	53.511(19.279-89.933)	395253.429(141494.343-675996.910)	81.041(28.945-139.183)	51.447(32.557-66.753)	1.470(1.384-1.555)
South Asia	7132.169(2640.926-11607.450)	1.531(0.563-2.476)	20677.894(7382.986-34848.403)	1.632(0.586-2.779)	6.613(-9.204-23.277)	0.020(-0.089-0.129)	274661.459(100118.189-456073.677)	46.647(17.115-77.024)	849535.236(291308.133-1466704.807)	55.968(19.283-95.917)	19.982(6.648-33.751)	0.368(0.284-0.453)
Central Sub-Saharan Africa	270.980(89.327-490.869)	1.365(0.459-2.421)	536.338(178.697-944.251)	1.103(0.374-1.962)	-19.214(-37.273-1.923)	-0.944(-1.086--0.802)	9703.067(3190.986-17542.669)	39.419(13.317-70.680)	24260.496(8360.503-41844.669)	37.180(12.809-63.997)	-5.680(-25.178-14.161)	-0.403(-0.536--0.270)
Eastern Sub-Saharan Africa	884.676(313.145-1513.476)	1.303(0.477-2.212)	1329.845(466.884-2274.554)	0.868(0.303-1.485)	-33.416(-41.684--23.053)	-1.646(-1.762--1.529)	29424.941(10245.573-49809.604)	36.283(12.881-61.558)	50980.976(17845.109-86361.738)	26.502(9.265-45.170)	-26.959(-36.008--16.835)	-1.344(-1.449--1.239)
Southern Sub-Saharan Africa	736.867(261.313-1235.808)	2.962(1.045-4.979)	1816.378(637.793-2990.106)	3.470(1.226-5.738)	17.162(3.191-32.963)	0.826(0.419-1.234)	23910.307(8373.615-39558.803)	85.189(29.948-141.559)	60898.367(21312.341-98935.685)	101.648(35.765-165.743)	19.320(6.405-32.113)	0.863(0.558-1.170)
Western Sub-Saharan Africa	656.932(229.044-1134.112)	0.841(0.295-1.434)	1489.023(513.811-2514.105)	0.866(0.299-1.474)	2.976(-13.387-22.402)	-0.064(-0.288-0.161)	23188.318(8211.360-39676.265)	25.146(8.952-42.841)	65205.068(23585.814-113620.664)	29.621(10.627-51.423)	17.799(1.238-35.610)	0.350(0.145-0.557)

Conversely, the age-standardized DALYs rate per 100,000 population increased from 36.329 (95% UI: 13.080-61.041) in 1990 to 42.523 (95% UI: 15.353-72.769) in 2021, representing a 17.049% increase (95% UI: 9.065% to 25.557%). The positive EAPC of 0.292 (95% CI: 0.217 to 0.368) further confirms this upward trend in disease burden.

### 3.2 Regional level burden and trends of T2DM-SHS

Our analysis revealed substantial heterogeneity in the burden of T2DM-SHS across different SDI quintiles and geographical regions. High SDI regions experienced the most significant reduction in ASMR, with a 44.596% decrease (95% UI: -47.869% to -41.383%) from 1990 to 2021, and an EAPC of -2.357 (95% CI: -2.578 to -2.135), indicating a rapid and consistent decline. However, these regions also observed a 9.797% increase (95% UI: -1.670% to 20.041%) in age-standardized DALYs rates, suggesting a shift towards a non-fatal disease burden. In contrast, low SDI regions showed more modest improvements, with a 7.150% reduction decrease in death rates (95% UI: -18.285% to 4.749%) and a 7.673% increase in DALYs rates (95% UI: -2.764% to 18.412%), highlighting persistent health challenges in resource-limited settings.

Regional analysis revealed varying trends across different areas. East Asia demonstrated significant progress, with a 19.173% reduction in death rates (95% UI: -36.044% to 2.613%) and a negative EAPC of -0.873 (95% CI: -1.104 to -0.642). However, the region also experienced a 14.787% increase in DALYs rates (95% UI: 0.021% to 29.201%). Sub-Saharan Africa showed mixed results, with Southern Sub-Saharan Africa experiencing a 17.162% increase in death rates (95% UI: 3.191% to 32.963%) and a 19.320% increase in DALYs rates (95% UI: 6.405% to 32.113%). The North Africa and Middle East region observed a slight 3.316% increase in death rates (95% UI: -12.027% to 14.776%) but a substantial 51.447% increase in DALYs rates (95% UI: 32.557% to 66.753%), indicating a significant rise in the non-fatal health burden in this region. These diverse regional trends underscore the complex nature of the global burden of T2DM-SHS.

### 3.3 National level burden and trends of T2DM-SHS

Our study examined the disease burden of T2DM-SHS across 204 countries, comparing data from 1990 and 2021 to identify significant changes in both case numbers and age-standardized rates. Country-specific analysis revealed diverse patterns. In China, for instance, case numbers increased from 5,548.24 (95% UI: 2,136.21-9,096.75) in 1990 to 12,627.68 (95% UI: 4,542.61-20,784.19) in 2021, while the age-standardized rate decreased from 0.77 (95% UI: 0.30-1.25) to 0.63 (95% UI: 0.23-1.04) per 100,000 population. Similar trends were observed in the Democratic People’s Republic of Korea and Taiwan, China The majority of countries experienced an increase in case numbers between 1990 and 2021. Although some countries showed reductions in incidence and death rates, the overall number of DALYs increased significantly. Interestingly, the percentage change from 1990 to 2021 revealed negative growth in age-standardized rates for most countries. For instance, China exhibited a change of -17.97% (95% UI: -35.83% to 6.17%), suggesting that despite an increase in case numbers, interventions targeting death and incidence rates have some effectiveness during this period.

### 3.4 Global and regional burden of T2DM-SHS

**Figure 1:**
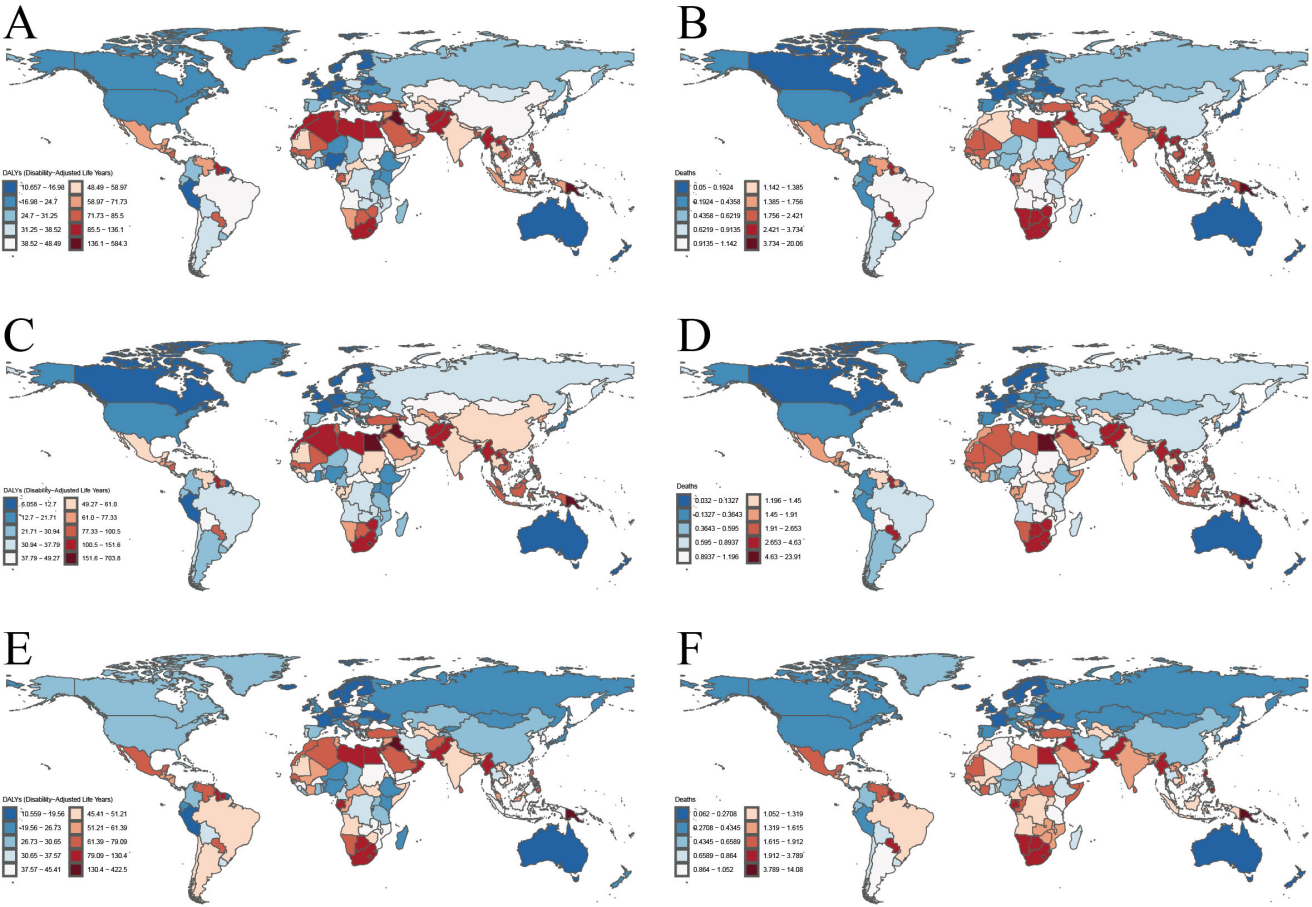
The age-standardized DALYs rates and ASMR for T2DM-SHS in 204 countries and territories in 2021. **(A)**. age-standardized DALYs rates for both sexes. **(B)** ASMR for both sexes. **(C)** age-standardized DALYs rates for female. **(D)** ASMR for female. **(E)** age-standardized DALYs rates for male. **(F)** ASMR for male.

Our analysis revealed significant geographical variations in the burden of T2DM-SHS across 204 countries and territories in 2021. As shown in **Figure 1**, the highest age-standardized DALYs rates for both sexes were observed in parts of Africa, the Middle East, and South Asia, with several countries reporting rates exceeding 71.73 per 100,000 population. In contrast, most high-income countries (HICs) in North America, Western Europe, and Australasia demonstrated lower rates, generally below 34.7 per 100,000 population. The distribution of ASMR largely mirrored the DALYs pattern, with the highest rates (>2.421 per 100,000) concentrated in similar regions. However, some countries, particularly in Sub-Saharan Africa and South Asia, showed disproportionately high death rates relative to their DALYs rates.

Gender-specific analysis revealed notable disparities. Females generally experienced higher burdens across most regions, particularly in terms of DALYs rates, with this disparity most pronounced in parts of Africa, the Middle East, and South Asia. For females, the highest DALYs rates (>77.33 per 100,000) and ASMR (>2.683 per 100,000) were observed in a larger number of countries compared to males. In contrast, male-specific maps showed a more moderate distribution of burden, with fewer countries in the highest categories for both DALYs and death rates.

Regionally, Sub-Saharan Africa, particularly the southern region, consistently showed high burdens across all metrics and for both genders. The Middle East and North Africa region demonstrated considerable heterogeneity, with some countries experiencing very high burdens while others showed moderate levels. East and Southeast Asia exhibited generally displayed moderate burdens, with notable exceptions of high-burden countries. HICs, including North America, Western Europe, and Australasia, consistently displayed the lowest burdens across all metrics.

### 3.5 Global and regional burden of T2DM-SHS

**Figure 2:**
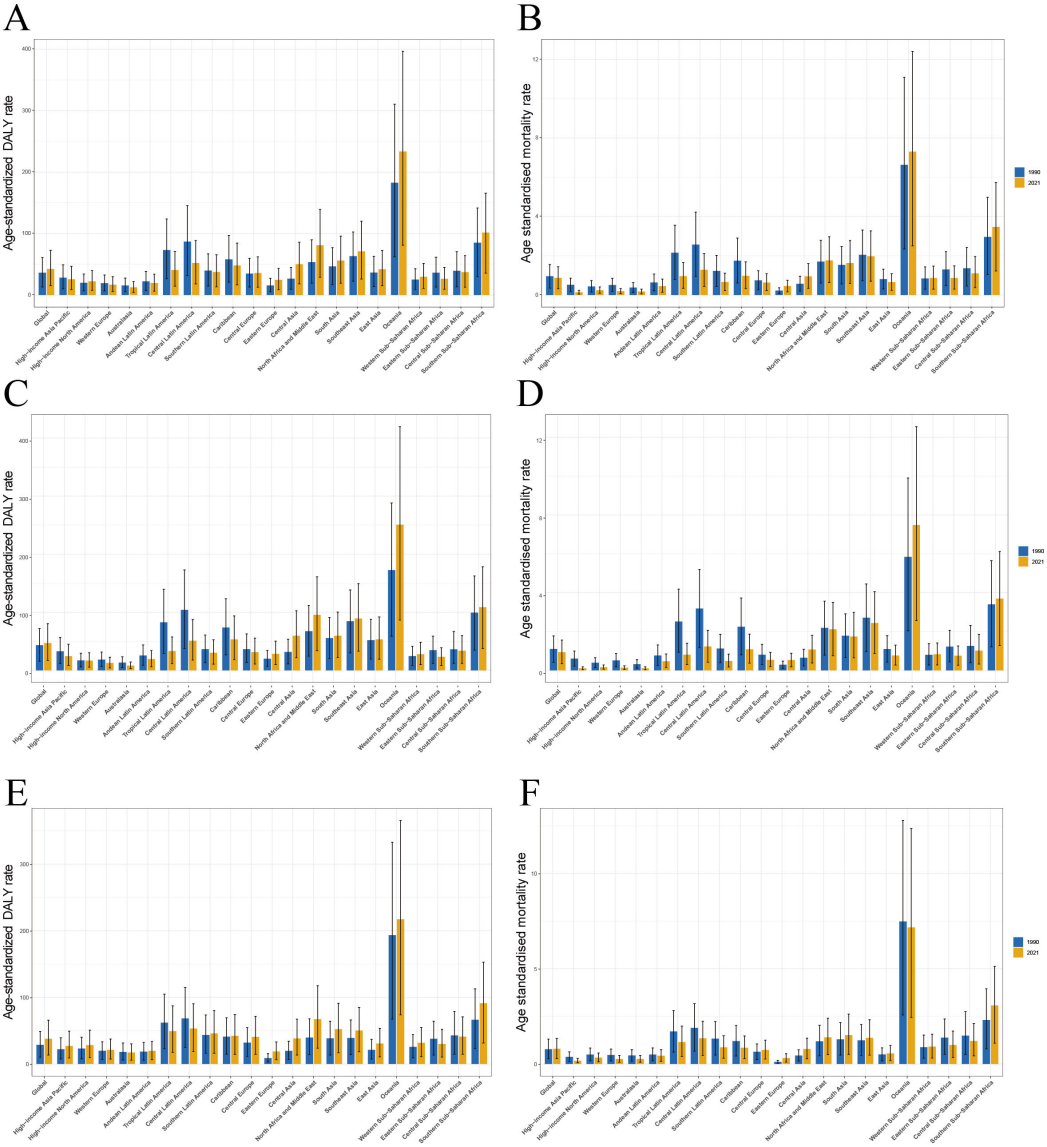
The age-standardized DALYs rates and ASMR for T2DM-SHS in Global and the 21 global burden of Disease regions in 2021. **(A)** age-standardized DALYs rates for both sexes. **(B)** ASMR for both sexes. **(C)** age-standardized DALYs rates for female. **(D)** ASMR for female. **(E)** age-standardized DALYs rates for male. **(F)** ASMR for male.

Our analysis of the GBD data for 2021 revealed significant variations in the burden of T2DM-SHS cross 21 global regions (**Figure 2**). The age-standardized DALYs rates and ASMR exhibited distinct patterns across different geographical areas and between genders.

Oceania consistently demonstrated the highest burden, with age-standardized DALYs rates exceeding 300 per 100,000 population and ASMR approaching 12 per 100,000 in 2021 for both sexes combined. Southern Sub-Saharan Africa and South Asia also exhibited high rates, with DALYs rates ranging between 100–150 per 100,000 and ASMRs around 4–5 per 100,000. In contrast, HICs such as Western Europe, Australasia, and High-income North America showed the lowest rates, with DALYs generally below 50 per 100,000 and ASMR below 1 per 100,000.

Gender-specific analysis revealed that females generally experienced higher burdens across most regions, particularly in terms of DALYs rates. This disparity was most pronounced in Oceania, Southern Sub-Saharan Africa, and South Asia. For instance, females in Oceania had a DALYs rate exceeding 350 per 100,000 and an ASMR approaching 14 per 100,000 in 2021, significantly higher than the rates for males in the same region. Male-specific data showed a more moderate distribution of burden, with lower peaks compared to females, especially in high-burden regions.

Temporal analysis comparing data from 1990 and 2021 revealed a general increase in both DALYs and death rates across most regions, with particularly pronounced rises in Oceania and parts of Sub-Saharan Africa. In contrast, some HICs showed minimal changes or slight decreases in burden over time, suggesting potential improvements in SHS exposure control or diabetes management. Notably, the gender gap in disease burden appeared to widen in several regions between 1990 and 2021, with females experiencing steeper increases in both DALYs and death rates.

**Figure 3:**
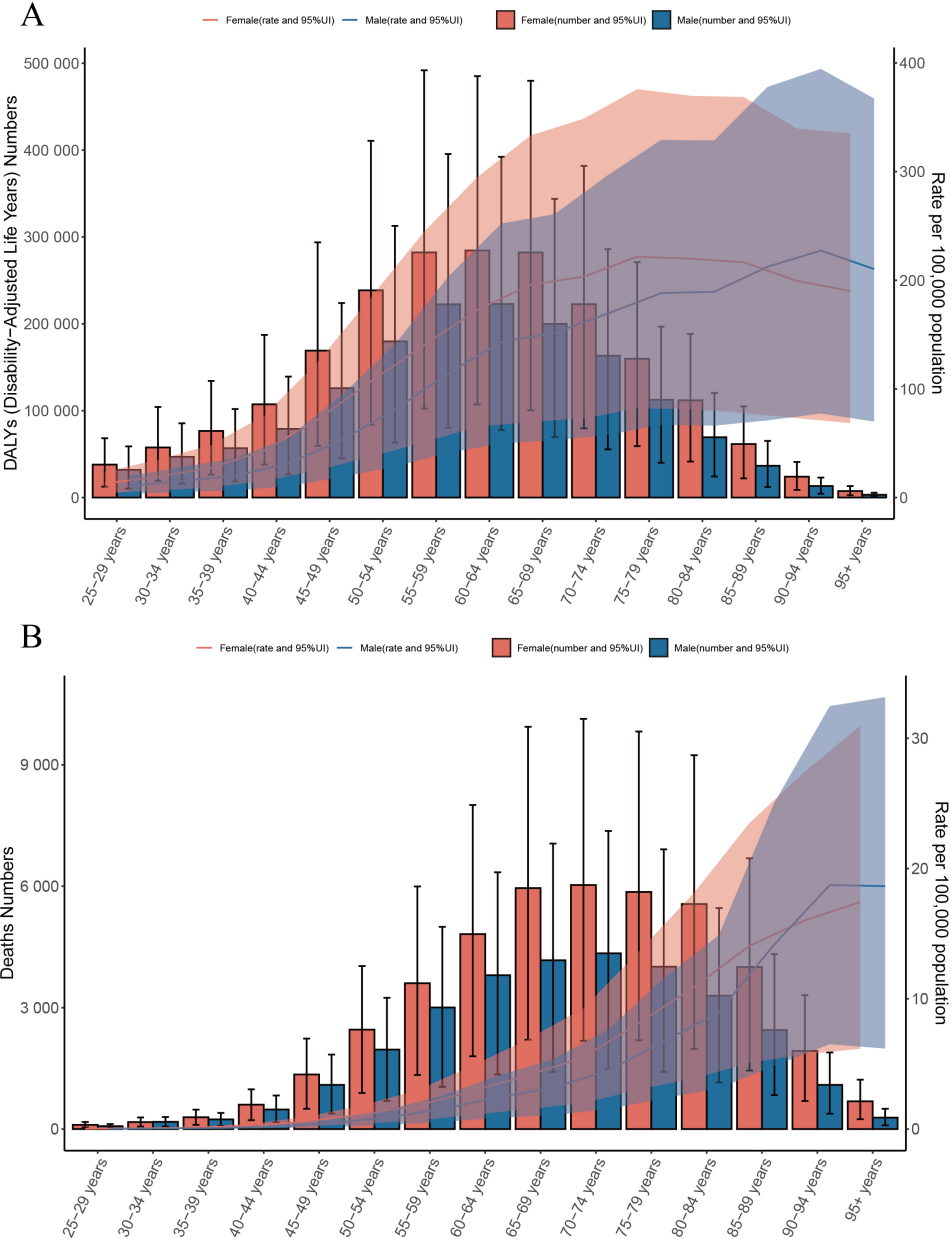
**(A)** Global number of DALYs and DALY rate of T2DM-SHS per 100,000 population, by age and sex, in 2021; DALYs=disability adjusted life years; **(B)** Global number of deaths and death rate of T2DM-SHS per 100,000 population, by age and sex, in 2021.

Our analysis of the global burden of T2DM-SHS in 2021 revealed significant age- and sex-specific patterns in both DALYs and deaths. The DALYs for females shows an increasing trend in the age group of 25–29 to 75–79 years, peaking in the 75–79 age group (**Figure 3A**). However, following this peak, there is a slowly decline in the DALYs rate as age continues to increase. For males, the DALYs rate peaked in the 90–94 age group, and then declined with further aging. The DALYs rate for females was consistently higher than that for males until the 90–94 age group, after which it became lower in females than in males. Similarly, the number of DALYs for both sexes increased with age, peaking in the 60–64 age group for both. Subsequently, the number of DALYs decreased with age. Notably, a significant increase in DALYs was evident from the 35–39 years age group onwards, indicating the onset of substantial T2DM-SHS burden in early middle age.

Regarding death, the number of deaths attributed to T2DM-SHS increased dramatically with age, peaking in the 70–74 years age group for both sexes (**Figure 3B**). Females consistently showed higher numbers of deaths across all age groups compared to males. ASMR per 100,000 population showed an exponential increase with age, starting with the 90–94 age group, and is higher in the male group. This shift in the leading results for the male and female groups is similar to that of the DALYs rate.

**Figure 4:**
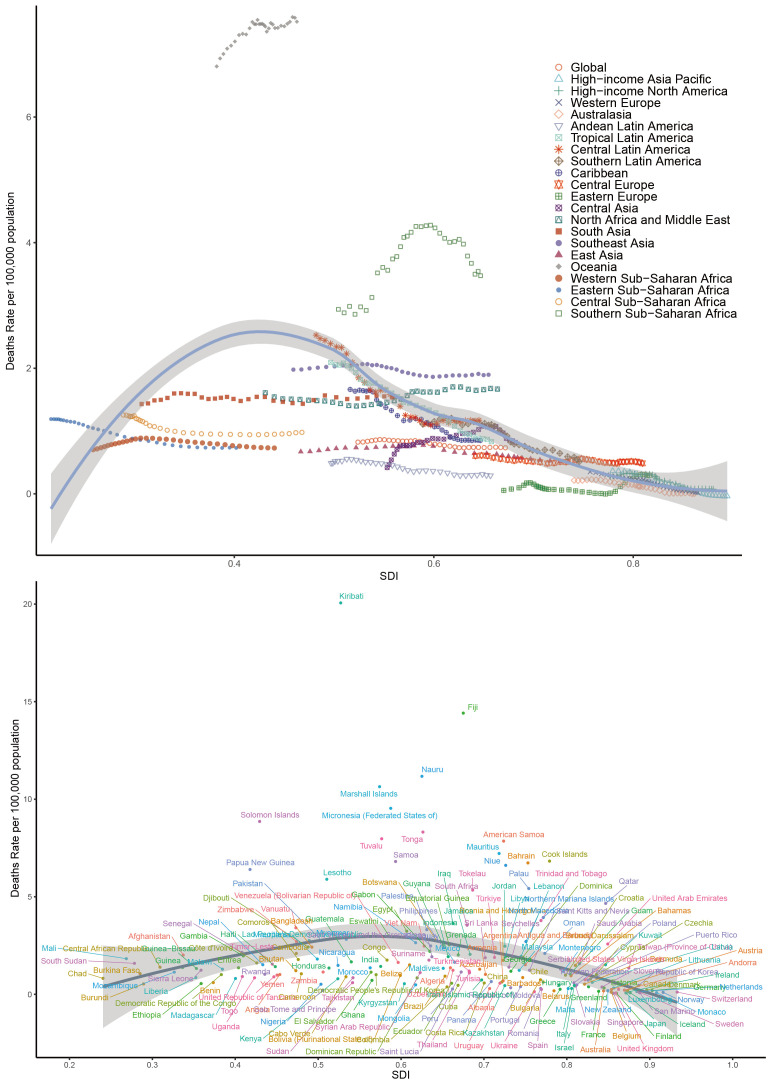
Age-standardized DALYs rates of T2DM-SHS for the 21 global burden of Disease regions and 204 countries and territories by SDI, 1990–2021.

Our analysis of the global burden of T2DM-SHS revealed several key findings across different SDI levels from 1990 to 2021. Globally, an inverted U-shape relationship was observed between SDI and age-standardized DALYs rates, with the highest rates occurring at mid-range SDI levels (**Figure 4**). HICs, including North America, Western Europe, and Asia Pacific, consistently demonstrated lower DALYs rates across the SDI spectrum. In contrast, Sub-Saharan African regions exhibited the highest DALYs rates, particularly at lower SDI levels. Latin American regions showed a significant decline in DALYs as SDI increased, while East Asia and Southeast Asia displayed unique patterns, with DALYs rates increasing as SDI improved, contrary to the global trend.

The global pattern of death rates closely mirrored that of DALYs, showing an inverted U-shape relationship with SDI (**Appendix Figure 4S** in **Supplementary Material**). Oceania emerged as an outlier, with exceptionally high death rates, particularly for countries like Kiribati, Fiji, and Nauru, despite their mid-range SDI values. HICs consistently maintained lower death rates across all SDI levels. Sub-Saharan African regions showed high variability in death rates, with some countries experiencing rates comparable to HICs despite lower SDI scores. In contrast, South Asia and Southeast Asia demonstrated a gradual decrease in death rates as SDI increased, aligning with the global trend.

**Figure 5:**
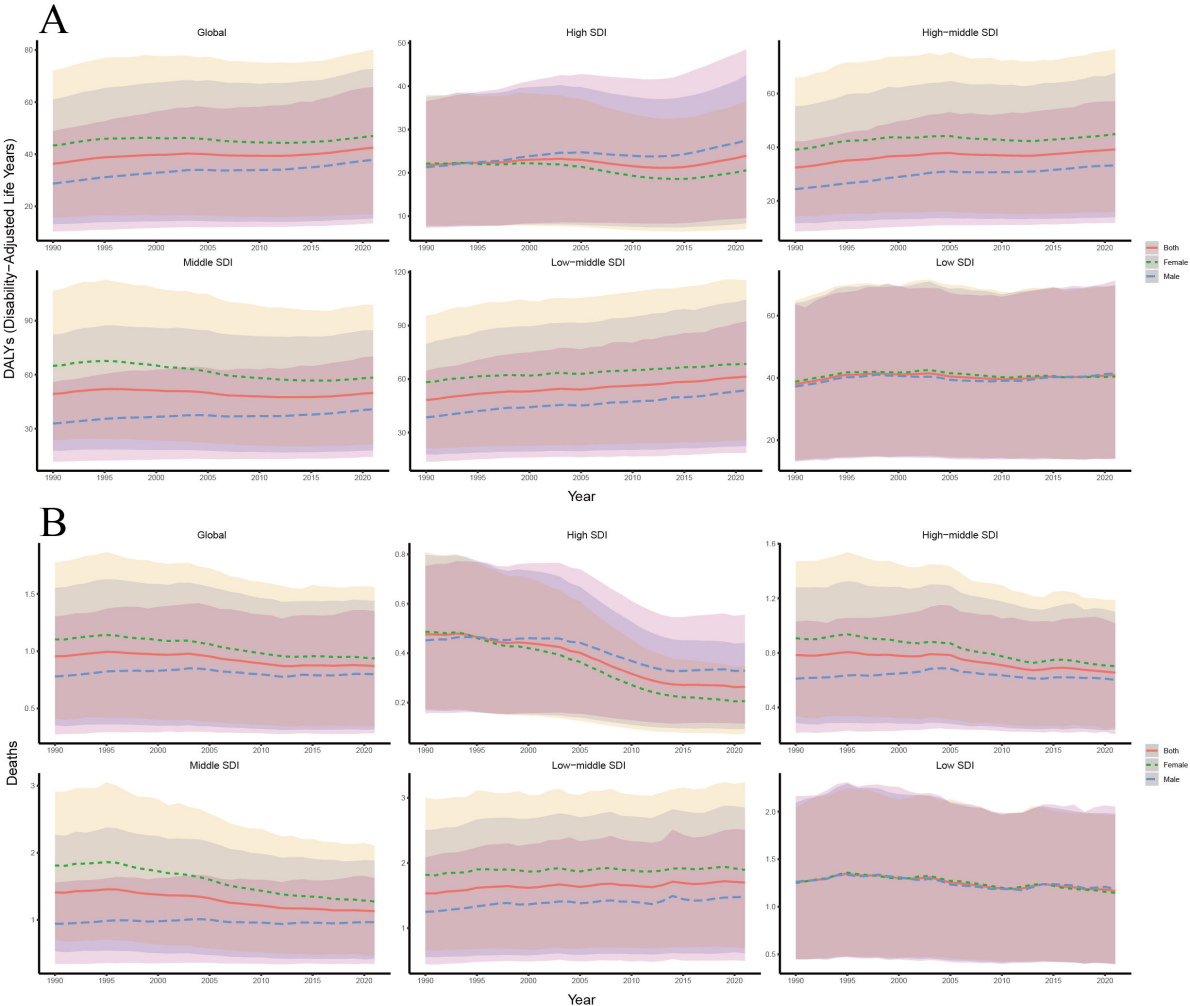
**(A)** Trends in age-standardized DALYs rates of T2DM-SHS from 1990 to 2021 by SDI and different sexes with 95% uncertainty intervals; **(B)** Trends in age-standardized death rates of T2DM-SHS from 1990 to 2021 by SDI and different sexes with 95% uncertainty intervals.

Our analysis of trends in age-standardized DALYs and death rates of T2DM-SHS from 1990 to 2021, stratified by SDI and sex, revealed several key findings (**Figure 5**). DALYs in high SDI regions have consistently been the lowest across all SDI categories. Gender-wise, DALYs for the Global population showed a slight increase over the period, with females consistently having higher DALYs than males. However, in contrast to the Global trend, female DALYs declined in both high and middle SDI regions. The gender disparity across SDI regions was also notable: except for high and low SDI regions, females had higher DALYs than males in most other regions. In 1990, female DALYs in high SDI regions were slightly higher than those of males, but since 1995, male DALYs in high SDI regions have surpassed those of females, a trend significantly different from the global pattern. In low SDI regions, the gender gap in DALYs was the smallest, with a trend emerging post-2015 where males had higher DALYs than females. Variations across SDI levels were notable: high SDI regions demonstrating a marked increase in DALY rates, particularly for females, while low SDI regions showed the highest overall rates but with a slight decrease over time.

From 1990 to 2021, the globally age-standardized death rate showed a slight decline, with female death rates consistently higher than those of males. In high SDI regions, death rates were the lowest and decreased significantly, with a sharper decline in females compared to males. Since 1995, females in high SDI regions have shown lower death rates than males, a trend similar to that observed in DALYs. In low SDI regions, the gender disparity in death rates was small, although both male and female death rates declined slightly over time. In contrast to the global trend, female death rates in low-middle SDI regions have been slowly increasing, paralleling the trend seen in DALYs for these regions.

The 95% uncertainty intervals for DALYs and death estimates were widest in low SDI regions, indicating greater variability and uncertainty in these areas. Comparing DALYs and death trends shows that while global DALYs slightly increased, death rates decreased slightly. This suggests that the disability burden from T2DM-SHS may still be rising in most regions, while middle SDI regions show the opposite trend.

## 4 Discussion

By addressing critical research gaps in understanding the global burden of T2DM-SHS, our study reveals several important findings. First, in most regions of the world, we found significant gender differences, with the burden generally higher for female than for male. Second, while deaths from T2DM-SHS have decreased globally, DALYs have increased, with DALYs being highest especially in old adults (typically defined as individuals aged 60 and older). Third, there are marked differences in profiles across SDI regions. These findings challenge existing paradigms and underscore the nuanced relationship between SHS exposure and T2DM risk.

While prior studies by Qin et al. (10) and Bai et al. (11) established the epidemiological link between SHS exposure and T2DM risk through meta-analytical and population-level approaches, our findings on gender-specific burden patterns reveal critical socio-structural determinants overlooked by these foundational studies. Building upon their biological risk frameworks, we demonstrate that traditional gender roles (domestic vs occupational exposure), healthcare accessibility gaps, and economic stratification collectively amplify female vulnerability, thereby explaining why standardized tobacco control measures alone inadequately address the disparities observed in our global analysis. This synthesis of biological mechanisms and social determinants is consistent with emerging calls for gender-responsive public health strategies in a previous study by Huang et al. (12).

Regarding gender differences, with female DALYs being much higher than male DALYs in most parts of the world. This epidemiological pattern reflects synergistic biological-environmental interactions. Mechanistic studies indicate estrogen-mediated CYP enzyme activation accelerates nicotine metabolism (13, 14), while sex-specific inflammatory responses amplify SHS-induced insulin resistance through distinct ceramide accumulation patterns and β-cell stress responses (15, 16). Females further demonstrate heightened metabolic susceptibility to tobacco-induced glucose dysregulation, evidenced by steeper dose-response relationships in glycemic parameter deterioration (17). Emerging pathophysiology models delineate sex-dimorphic metabolic dysregulation via SHS-triggered LXRα/IGF-1 axis perturbations, synergistically driving T2DM progression through lipid-glucose homeostasis disruption (14, 18). Environmental cofactors amplify this vulnerability: prolonged domestic SHS exposure in low- and middle-income countries (LMICs) disproportionately affects females, particularly in household settings, despite lower rates of active smoking (19). Biomarker studies confirm pervasive exposure to SHS among pregnant females in developing regions, reflecting the different socio-cultural emphasis on smoke-free policies, and that female exposure to SHS is strongly influenced by socio-cultural influences (20, 21).

Our age-stratified analyses demonstrated a marked age-dependent increase in disease burden, with DALYs rates reaching their apex in the 75–79-year age group for females and the 90–94-year age group for males. This heightened vulnerability among older adults can be attributed to several interconnected mechanisms. Primarily, longitudinal evidence indicates that chronic SHS exposure significantly amplifies cardiovascular risk and impairs glucose homeostasis in older adults. This is exemplified by a comprehensive Korean cohort study, which demonstrated that cumulative SHS exposure was independently associated with progressive deterioration of glucose metabolic parameters, particularly among older adults (22). Furthermore, extensive epidemiological data suggest that passive smoking substantially elevates the risk of diabetic complications in the older adults, with significant increases in microangiopathy and neuropathy incidence (23).

Exposure to SHS amplifies the chronic low-grade inflammatory state driven by immunosenescence in older adults with multimorbidity, activating the NF-κB pathway and thereby exacerbating insulin resistance (24). This interaction is compounded by polypharmacy-induced metabolic burden (17) and behavioral-structural vulnerabilities: only 15% of elderly passive smokers meet recommended exercise levels, while 40% exhibit obesity, creating a “metabolic-behavioral” dual risk (25). Socioeconomically disadvantaged individuals face additional barriers, including limited healthcare access and structural challenges (e.g., multigenerational households or care facilities), which perpetuate SHS exposure and escalate health risks.

In addition to individual-level risk factors, socioeconomic circumstances directly shape the burden of T2DM-SHS. Our analysis reveals an inverted U-shaped relationship between SDI and T2DM-SHS burden, with high SDI countries exhibiting the lowest DALYs and deaths. Three mechanisms explain this disparity: First, comprehensive smoke-free legislation in high SDI regions reduces public SHS exposure (26). Second, low/middle SDI regions face healthcare constraints (e.g., limited insulin access and screening programs), exacerbating SHS-driven diabetes through delayed detection/intervention (27). Third, middle SDI regions’ delayed public health infrastructure development—particularly lagging tobacco control—amplifies SHS exposure despite economic growth (28).

Our analysis identifies Eastern Europe/Central Asia as the only region with male-predominant T2DM-SHS burden, contrasting global female predominance. This divergence stems from synergistic male-specific risks: culturally-prevalent heavy alcohol use and industrial sector occupational exposures interacting with SHS to accelerate metabolic dysfunction (12, 29).

Despite declining T2DM-SHS mortality from SHS reduction efforts, rising T2DM-SHS DALYs reflects newly recognized pathophysiological cascades: SHS-induced oxidative stress directly impairs β-cell function (30) while exacerbating lipid dysregulation in vulnerable subgroups (31). This dual mechanism emphasizes the need for interventions addressing both exposure mitigation and metabolic restoration. For LMICs navigating socioeconomic transitions, we advocate a stratified intervention framework: (1) Infrastructure reinforcement integrating SHS screening into primary diabetes care and deploying community-based prevention programs (32); (2) Policy adaptation of evidence-backed strategies like tobacco taxation and smoke-free zoning, calibrated to local contexts (29); and (3) Vulnerability-targeted protection through strongly recommended primary care risk assessments and equitable access to air quality monitoring technologies. Crucially, sustained public health education via institutional channels (33) must synergize with cross-sector collaboration among clinicians, policymakers, and community stakeholders to align diabetes prevention with SHS control. This tripartite approach – simultaneously targeting exposure pathways, healthcare system gaps, and socioeconomic drivers – provides a scalable model to address T2DM-SHS, emphasizing context-specific implementation while preserving biological plausibility across diverse populations. This duality necessitates interventions targeting both exposure reduction and metabolic rescue pathways.

Our study has some limitations. The burden of T2DM-SHS may be systematically underestimated in low SDI areas due to severe resource constraints and diagnostic limitations. Methodological challenges include: (1) Exposure assessment bias: Self-reported SHS exposure itself is susceptible to recall bias, social desirability effects, and subjective perceptions. Available evidence suggests that large discrepancies between self-reported and objectively measured exposures can lead to significant underestimation of health effects (10, 34, 35). (2) Complexity of confounders: Inadequate control for potential confounders may mask the independent effects of SHS on T2DM. Multifactorial influences such as physical activity, dietary patterns, socioeconomic status, mental health, environmental exposures, and gene-environment interactions may modulate both SHS exposure and T2DM risk (11, 36). To address these limitations, in future studies, we can take the following steps: (1) adopt objective exposure assessment techniques (e.g., biomarker analysis); (2) utilize comprehensive multi-source data collection strategies; (3) develop complex statistical models that incorporate control for a large number of confounding factors.

## 5 Conclusion

Our comprehensive global analysis of T2DM-SHS reveals a complex landscape of disease burden characterized by significant gender, age, and socioeconomic disparities. The study demonstrates a paradoxical trend of decreasing mortality but increasing DALYs, with females experiencing disproportionately higher burdens, particularly in Oceania, Southern Sub-Saharan Africa, and South Asia. An inverted U-shaped relationship between SDI and disease burden underscores the nuanced interplay of biological, environmental, and socioeconomic factors. These findings urgently call for context-specific, targeted interventions that address the multifaceted mechanisms of SHS-induced metabolic vulnerability, with a critical focus on protecting vulnerable populations, particularly female and old adults in low- and middle-SDI regions.

## Data Availability

Publicly available datasets were analyzed in this study. This data can be found here: https://www.healthdata.org/research-analysis/gbd.
