# Supplementary material for: Global burden of type 2 diabetes attributable to secondhand smoke: a comprehensive analysis from the GBD 2021 study
Source: Front Endocrinol (Lausanne). 2025 Apr 29;16:1506749. doi: 10.3389/fendo.2025.1506749 (PMC12069062; doi:10.3389/fendo.2025.1506749)
Supplement: Supplementary file 1 [file DataSheet1.docx]

**Appendix**


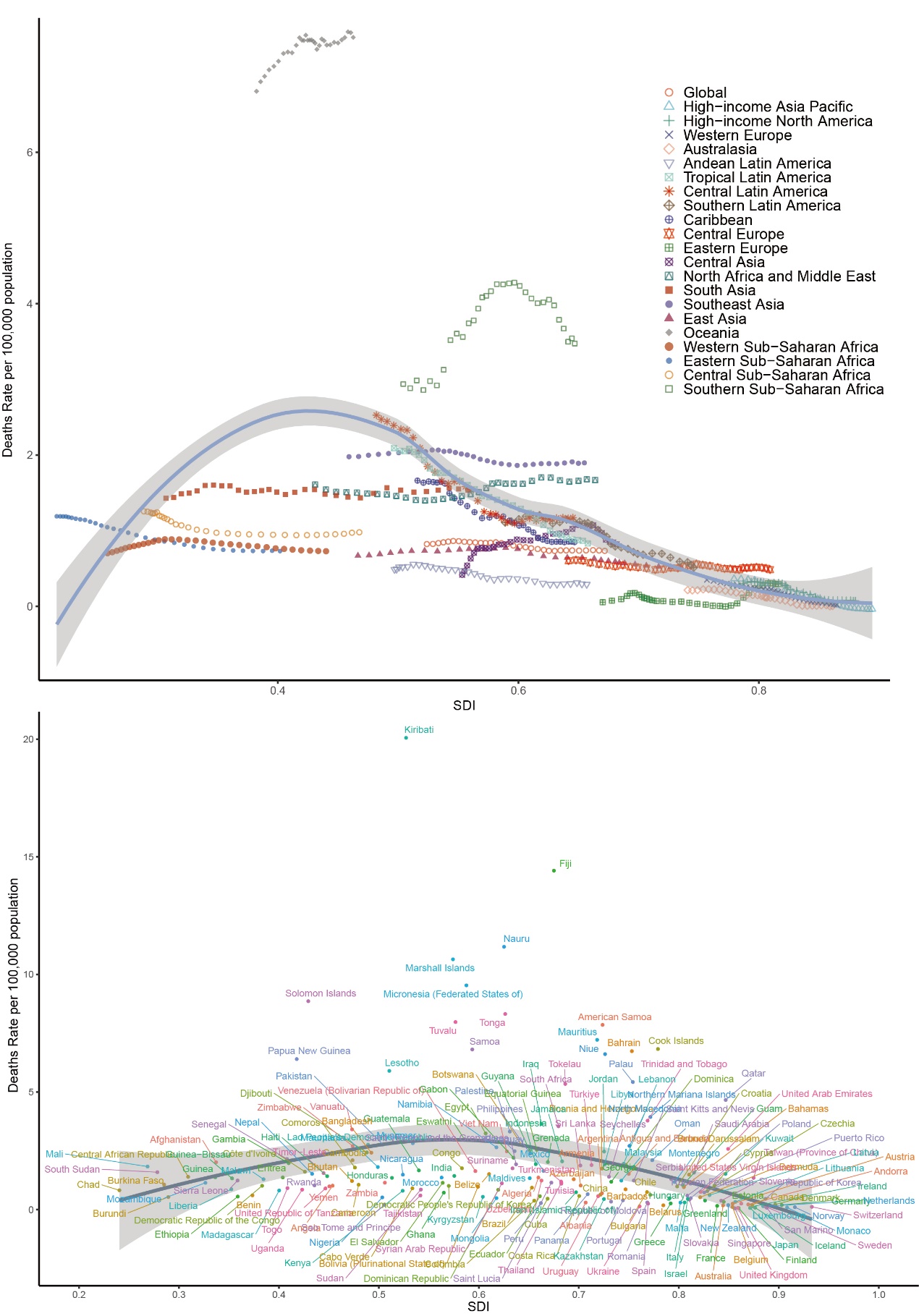


**Figure 4S：**Death rates of T2DM-SHS for the 21 global burden of Disease regions and 204 countries and territories by socio-demographic index (SDI) , 1990–2021.
